# Supplementary material for: The therapeutic effects of gingival mesenchymal stem cells and their exosomes in a chimeric model of rheumatoid arthritis
Source: Arthritis Res Ther. 2023 Oct 26;25:211. doi: 10.1186/s13075-023-03185-6 (PMC10601129; doi:10.1186/s13075-023-03185-6)
Supplement: Supplementary file 1 — Additional file 1: Supplemental Table 1. GMSC Home to the Site of Implantation. Supplemental Figure 1. GMSC and GMSCExo Reduce RASF MMP-14 Activity In Vitro Co-incubation of RASF with GMSCExo significantly reduces the RASF derived MMP-14 activity, *p < 0.05. [file 13075_2023_3185_MOESM1_ESM.docx]

|  | V405 Positive Cells | V405 Positive Frequency |
| --- | --- | --- |
| Cartilage Implant | 2705 | 1.23% |
| Blood | 0 | 0.00% |
| Kidney | 85 | 0.02% |
| Liver | 5 | 0.00% |
| Lung | 1949 | 0.22% |

**Supplemental Table 1. GMSC Home to the Site of Implantation**


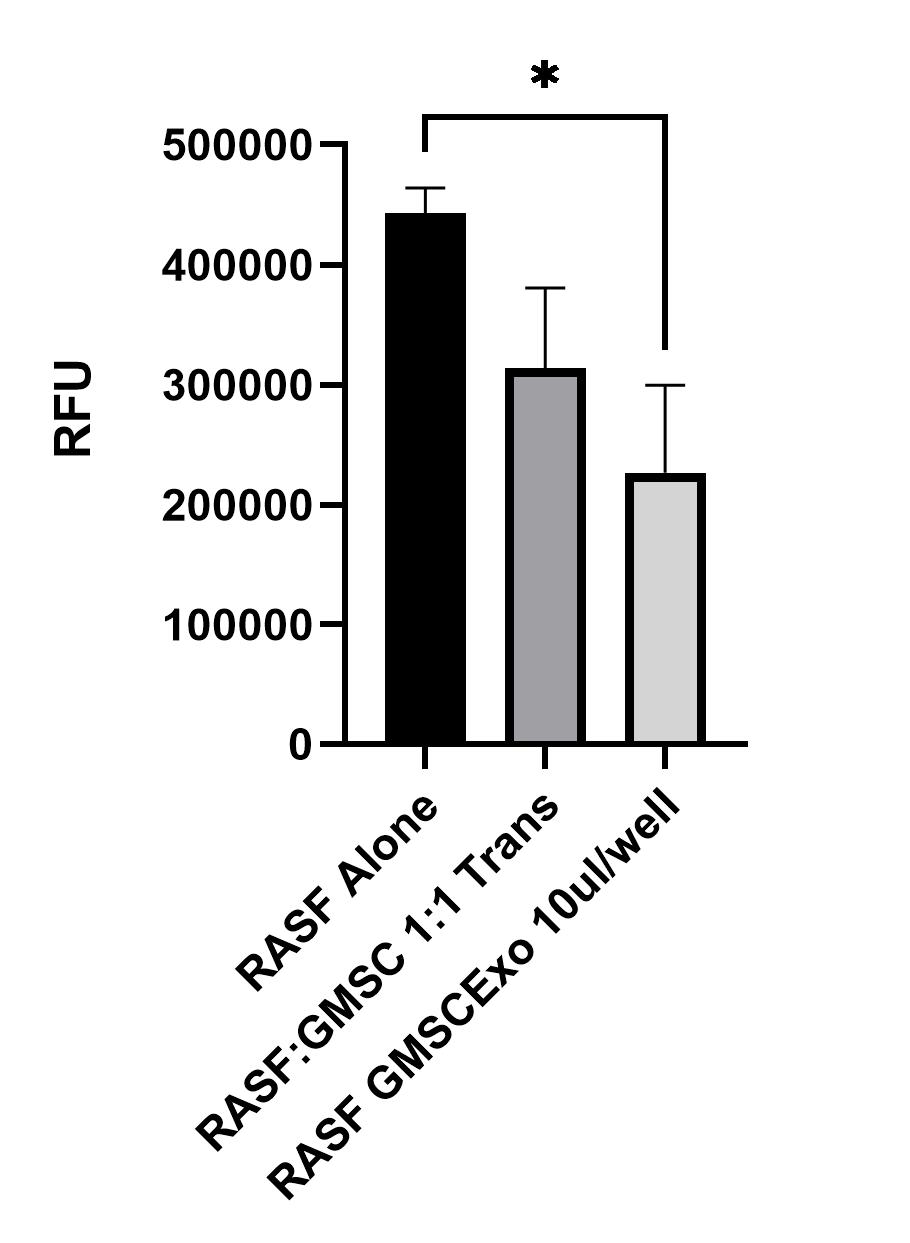


**Supplemental Figure 1. GMSC and GMSCExo Reduce RASF MMP-14 Activity I*n Vitro***

Co-incubation of RASF with GMSCExo significantly reduces the RASF derived MMP-14 activity, *p<0.05.
